# Strain-specificity in the hydrogen sulphide signalling network following dietary restriction in recombinant inbred mice

**DOI:** 10.1007/s11357-020-00168-2

**Published:** 2020-03-11

**Authors:** Stephen E. Wilkie, Lorna Mulvey, William A. Sands, Diana E. Marcu, Roderick N. Carter, Nicholas M. Morton, Christopher Hine, James R. Mitchell, Colin Selman

**Affiliations:** 1 Glasgow Ageing Research Network (GARNER), Institute of Biodiversity, Animal Health and Comparative Medicine, College of Medical, Veterinary and Life Sciences, University of Glasgow, Glasgow, G12 8QQ UK; 2 Molecular Metabolism Group, University/BHF Centre for Cardiovascular Sciences, Queens Medical Research Institute, University of Edinburgh, Edinburgh, EH16 4TJ UK; 3 Department of Cardiovascular and Metabolic Sciences, Cleveland Clinic Lerner Research Institute, Cleveland, OH 44195 USA; 4 Department of Genetics and Complex Diseases, Harvard T.H. Chan School of Public Health, Boston, MA 02115 USA

**Keywords:** Caloric restriction, Ageing, ILSXISS, Longevity, Sulphide, Dietary restriction, 3-mercaptopyruvate sulfurtransferase

## Abstract

Modulation of the ageing process by dietary restriction (DR) across multiple taxa is well established. While the exact mechanism through which DR acts remains elusive, the gasotransmitter hydrogen sulphide (H_2_S) may play an important role. We employed a comparative-type approach using females from three ILSXISS recombinant inbred mouse strains previously reported to show differential lifespan responses following 40% DR. Following long-term (10 months) 40% DR, strain TejJ89—reported to show lifespan extension under DR—exhibited elevated hepatic H_2_S production relative to its strain-specific ad libitum (AL) control. Strain TejJ48 (no reported lifespan effect following 40% DR) exhibited significantly reduced hepatic H_2_S production, while H_2_S production was unaffected by DR in strain TejJ114 (shortened lifespan reported following 40% DR). These differences in H_2_S production were reflected in highly divergent gene and protein expression profiles of the major H_2_S production and disposal enzymes across strains. Increased hepatic H_2_S production in TejJ89 mice was associated with elevation of the mitochondrial H_2_S-producing enzyme 3-mercaptopyruvate sulfurtransferase (MPST). Our findings further support the potential role of H_2_S in DR-induced longevity and indicate the presence of genotypic-specificity in the production and disposal of hepatic H_2_S in response to 40% DR in mice.

**Electronic supplementary material:**

The online version of this article (10.1007/s11357-020-00168-2) contains supplementary material, which is available to authorized users.

## Introduction

Empirical evidence has existed for over a century that dietary restriction (DR) increases lifespan and healthspan across multiple species (Fontana and Partridge 2015; Picca et al. 2017; Weindruch and Walford 1988). In mice, significant strain-specific differences in lifespan exist (Turturro et al. 1999; Yuan et al. 2009) and genetic background may consequently play an important but under-appreciated role in how particular strains respond to DR (Hempenstall et al. 2010; Ingram and de Cabo 2017; Mitchell et al. 2016; Mulvey et al. 2014; Selman and Swindell 2018; Swindell 2012). For example, two independent studies have reported that recombinant inbred ILSXISS mice show significant strain-specificity in longevity following 40% DR (Liao et al. 2010; Rikke et al. 2010), and phenotypic parameters linked to the ageing process, such as in mitochondrial function and adiposity, have been shown to differ between ILSXISS strains under 40% DR (Liao et al. 2011; Mulvey et al. 2016).

Precisely how DR facilitates its beneficial effects on lifespan and healthspan has proved challenging to elucidate, although many mechanisms have been proposed (Fontana and Partridge 2015; Hine and Mitchell 2015; Kennedy et al. 2007; Mair and Dillin 2008; Masoro 2005). One such putative mechanism is the gasotransmitter hydrogen sulphide (H_2_S). Direct manipulation of H_2_S levels through genetic, pharmacological or environmental means can modulate lifespan in invertebrate models (Hine and Mitchell 2015; Miller and Roth 2007; Qabazard et al. 2013; Shaposhnikov et al. 2018; Wei and Kenyon 2016) and elevated hepatic H_2_S production appears to be a conserved phenotype in long-lived mouse models, including DR and various genetic mutants (Hine et al. 2017; Mitchell et al. 2016). Pharmacological elevation of H_2_S has also been shown to ameliorate age-associated atherosclerosis, fibrosis, cognitive decline and kidney dysfunction in rodents (Das et al. 2018; Lee et al. 2018; Zhan et al. 2018), and partially rescued a progeroid phenotype in Werner syndrome fibroblasts (Talaei et al. 2013) and senescence in endothelial cells (Latorre et al. 2018). Furthermore, DR-induced protection from ischemia-reperfusion injury was abrogated in mice treated with an inhibitor of cystathionase-γ-lyase (CSE), the major hepatic H_2_S-producing enzyme (Hine et al. 2015), and longevity in mice following methionine restriction was associated with increased H_2_S production and a reduction in various senescence markers within the kidney (Wang et al. 2019). Consequently, it has been proposed that elevation of endogenous H_2_S may play a prominent role in the lifespan and healthspan effects of DR (Hine and Mitchell 2015).

Here, we employed a comparative-type approach (Mulvey et al. 2016) in which we determined hepatic and kidney H_2_S production, and hepatic transcript and protein levels of key enzymes involved in H_2_S metabolism in female mice from three genetically distinct recombinant inbred ILSXISS strains exposed to long-term (10 months) 40% DR. These strains have previously been reported to show variable lifespan responses to 40% DR ranging from life extension to life shortening relative to strain-specific ad libitum controls (Liao et al. 2010; Rikke et al. 2010).

## Methods

### Animals

The ILSXISS strains TejJ89, TejJ48 and TejJ114 were purchased from the Jackson Laboratory (Bar Harbour, Maine, URL: http://www.informatics.jax.org) as breeding pairs and experimental cohorts subsequently bred at The University of Glasgow. As previously discussed (Mulvey et al. 2016), female mice from strains TejJ89, TejJ48 and TejJ114 showed repeatable directional effects (TejJ89 lifespan extension under dietary restriction (DR), TejJ48 lifespan unaffected under DR, TejJ114 lifespan shortening under DR) on lifespan following 40% DR across 2 independent studies, but that no strain-specific differences in lifespan were reported when these mice were maintained on an ad libitum (AL) diet (Liao et al. 2010; Rikke et al. 2010). It should be noted that several potential shortcomings to the experimental design of these original studies have been raised (Selman and Swindell 2018), not least that 40% DR may simply be sub-optimal in TejJ48 and TejJ114, and that lifespan extension in these strains is likely to be seen at a higher or lower level of DR; these dose-response experiments are still to be undertaken (Selman and Swindell 2018). However, for the purposes of this study, we were interested in whether there was a relationship between H_2_S production and reported lifespan following 40% DR. Female mice were used for all experiments because lifespan was only determined in female mice across both original studies (Liao et al. 2010; Rikke et al. 2010). In addition, we also examined components of the H_2_S signalling network in female C57BL/6J mice that followed a similar long-term 40% DR protocol, to further examine potential strain-specific effects. It has previously been shown that hepatic H_2_S production is increased female C57BL/6J mice under 40% DR (Mitchell et al. 2016).

All mice were maintained from weaning onwards at 22 ± 2 °C and on a 12L/12D cycle (lights on 0700–1900 h) in groups of 4 mice within shoebox cages (48 cm × 15 cm × 13 cm), with AL access to water and standard chow (CRM(P), Research Diets Services, LBS Biotech, UK; Atwater Fuel Energy-protein 22%, carbohydrate 69%, fat 9%). 10% DR was introduced in a graded fashion from 10 weeks of age and then held at 40% DR from 12 weeks onwards, with the food intake of the DR cohorts adjusted each week relative to the average weekly AL food intake of the appropriate age-matched and strain-specific AL controls (Hempenstall et al. 2010; Mulvey et al. 2016). Following 10 months of 40% DR (13 months of age) female mice were fasted overnight and culled using cervical dislocation under a UK Home Office Project Licence (60/4504) and following the “principles of laboratory animal care” (NIH Publication No. 86-23, revised 1985). Tissues were immediately dissected out, snap-frozen in liquid nitrogen and stored at − 80 °C until use.

### Hydrogen sulphide (H_2_S) production

Measurement of H_2_S production was performed in liver and kidney homogenates according to a previously described protocol (Hine and Mitchell 2017). Briefly, 100 mg of flash-frozen liver and kidney were lysed in passive lysis buffer. Protein concentration was determined by BCA assay (G Biosciences, MO, USA) and 100 μg of protein lysate was loaded into 96-well plate. A 150-μL reaction solution containing 10 mM L-cysteine and 1 mM pyridoxal-5′-phosphate was added to the protein lysate. Filter paper that had previously been cut to the size of the plate, soaked in 20 mM lead(II)acetate trihydrate for 20 min, then dried under vacuum, was then securely attached to the plate. The assembled plate was incubated at 37 °C for 1 h. H_2_S sulphide gas produced during this time collects in the head space between the top of the solution in the well and the lead(II)acetate paper, forming a brown-black substrate on the paper. The amount of H_2_S present in each sample was subsequently quantified by densitometry analysis (ImageJ) of the brown-black substrate.

### RNA extraction

RNA was isolated from liver tissue by addition of 500 μL of TRIzol (Life Technologies, USA) and subsequently homogenised using a glass-glass homogeniser. Samples were transferred to screw top Eppendorf tubes and 150 μL of chloroform added. Samples were then spun by centrifuge at 8000*g* and the supernatant containing the RNA isolate was taken to a fresh Eppendorf. RNA cleanup was performed according to instructions provided in RNAeasy Mini Kit (Qiagen, Germany), including the optional DNase digestion step.

### Reverse transcriptase quantitative-PCR

First strand synthesis of cDNA was performed by incubating 2 μg of RNA (quantified by spectrophotometry using Nanodrop 1000 UV-Vis spectrophotometer, ThermoScientific, MA, US) with 1 μg Random Primer Mix (Invitrogen) in a total volume of 15 μL with RNAse-free water at 70 °C for 5 min using a MJ research PTC-200 Peltier Thermal Cycler (Biorad, CA, US). Synthesis of cDNA was then performed by adding 10 μL of master mix (1 μL Promega M-MLV reverse transcriptase, 2μL Promega M-MLV 5x buffer, 5 μL pooled 10 mM dNTPs, 0.625 μL RNAseOUT 40 units/μL and 1.375 μL nuclease free water) to the first stand sample and heating to 37 °C for 1 h. Samples were then diluted 1:1 with PCR-grade water and used directly for RT-qPCR. RT-qPCR was performed in a 384-well PCR plate. Each well contained 1 μl of cDNA, 0.25 μL 10 mM upper primer, 0.25 μL 10 mM lower primer, 3.5 μL of PCR-grade water and 5 μL of QuantiFast SYBR green PCR 2x master mix (Qiagen, UK). PCR reaction was performed using a 7900HT Fast Real-Time PCR System (Applied Biosystems, CA, US). PCR profile was as follows: 95 °C for 5 min; 94 °C for 30 s, 60 °C for 30 s, 72 °C for 30 s for 40 cycles; 72 °C for 5 min. The endogenous control gene was β2M, which has been previously shown to be an appropriate housekeeping control gene for mouse dietary restriction studies (Gong et al. 2016). Gene expression was calculated by subtracting the Ct value for β2M from the Ct value pertaining to the gene of interest in each sample. As such, a lower ΔCt indicates a higher relative gene expression of mRNA transcripts and vice versa. Primer sequences are provided in Table S1.

### Western blotting

Protein lysate was obtained by homogenisation of liver tissue in 1 mL of ice cold RIPA buffer (Radio Immunoprecipitation Assay Buffer; 150 mM sodium chloride, 1% NP-40 or Triton X-100, 0.5% sodium deoxycholate, 0.1% sodium dodecyl sulphate, 50 mM Tris, pH 8) containing protease and phosphatase inhibitors (Halt™ Protease and Phosphatase Inhibitor Cocktail, Thermo Fisher Scientific, UK; phenylmethylsulfonyl fluoride, Sigma Life Sciences, Germany; Complete Mini EDTA-free protease inhibitor cocktail, Merck, NJ, US) using a glass-glass homogeniser. Homogenates were kept on ice for 40 min and then spun by centrifuge at 8000*g* for 10 min at 4 °C. The supernatant was collected and used as protein lysate. Protein concentration was assessed by BCA assay (G Biosciences, MO, USA) and 20 μg of protein was loaded per well into homemade 4–12% bis-tris polyacrylamide gels. Precision Plus Protein^TM^ Dual Xtra Standards protein marker (BioRad, CA, US) were added to a well on each gel. Proteins were separated by electrophoresis at 90 V for 90 min and then transferred onto nitrocellulose membrane at 0.25 V for 1 h. Membranes were stained with Ponceau-S (Sigma Life Sciences, Germany), briefly washed in deionised water and the resulting total protein stain was captured using a Chemidoc^TM^ XRS System (BioRad, CA, US). The Ponceau-S stain was removed by using 1xTBST (Tris-Buffered Saline Tween^20^) and the membrane was blocked with 5% milk in 1xTBST for 40 min. The membrane was washed 5 times with 1xTBST for 5 min under constant shaking. Primary antibodies (AbCam, Cambridge, UK) were added to the membrane in 5% BSA in 1xTBST. CSE (ab151769) primary antibody was used at 1:1000 dilution; CBS (ab135626) and MPST (ab85377) were used at 1:100. Primary antibodies were allowed to incubate with the membrane overnight at 4 °C, under constant shaking. HRP-linked anti-rabbit antibody (#7074; Cell Signalling Technology, London, UK) was used at 1:2000 dilution in 5% BSA in 1xTBST as the secondary antibody for all blots. The secondary antibody was allowed to incubate with the membrane for 1 h, under constant shaking. The membrane was washed 5 times with 1xTBST for 5 min, under constant shaking before addition of all antibodies and before imaging. For imaging, membranes were coated with Clarity™ Western ECL substrate (BioRad, CA, US) reagent and left to react for approx. 3 min before an image was developed under chemiluminescent conditions using a ChemiDoc™XRS System. Protein signals were quantified using densitometry software (ImageStudio; LiCor, NE, US) and normalised to the total protein signal of their respective lane.

### 3-Mercaptopyruvate sulfurtransferase activity assay

3-Mercaptopyruvate sulfurtransferase (MPST) activity was determined in liver by measuring thiocyanate production capacity as described previously for thiosulfate sulfurtransferase (TST) rhodanese activity (Morton et al. 2016), except that sodium 3-mercaptopyruvate (3-MP) was used as a substrate instead of sodium thiosulfate. In a 96-well plate, 20 μg of protein lysate in RIPA was mixed with 10 μL 200 mM 3-MP (Santa-Cruz, UK) and taken to 90 μL with 500 mM potassium phosphate pH 5.5 buffer. Samples were incubated at 37 °C for 2 min before addition of 10 μL 500 mM potassium cyanide. A calibration curve of 50, 25, 10, 5, 2.5, 1, 0.5, 0.25 and 0.1 mM potassium thiocyanate solutions was also prepared and exposed to the same conditions as above, excluding the addition of potassium cyanide. The reaction was allowed to occur for 5 min at 37 °C before termination by addition of 11 μL of 38% formaldehyde to all wells. Thiocyanate production was visualised by addition of 125 μL Fe(NO_3_)_3_/26% HNO_3_ where an orange-brown solution formed. Results were quantified by measuring absorbance for 460 nm light in a spectrophotometer (Multiscan GO Microplate Spectrophotometer, Thermo Scientific, MA, USA). All samples were performed in duplicate and the average 460 nm absorbance was calculated.

### Statistical analysis

All statistical analyses were performed using SPSS® Version 25 (IBM®, New York, USA) and Prism 6 (GraphPad Inc., La Jolla, USA) software. All data were analysed using a general linear modelling approach with treatment (AL or DR) and genotype (TejJ89, TejJ48 and TejJ114 (and where indicated, C57BL/6J)) introduced as fixed factors, and a post hoc Bonferroni test used for multiple comparisons. In all cases, non-significant interactions (*p >* 0.05) within the GLM analyses were removed in order to obtain the best-fitting model, with only significant interactions reported. All data were analysed by Grubbs outlier test with alpha set to 5%. Unless otherwise described, all results are presented as mean ± standard error of the mean (SEM), with *p* < 0.05 regarded as statistically significant. * denotes *p* < 0.05, ** denotes *p* < 0.01 and *** denotes *p* < 0.001.

## Results

### Genotype-specific hepatic H_2_S production following 40% DR in female ILSXISS mice

**Fig. 1:**
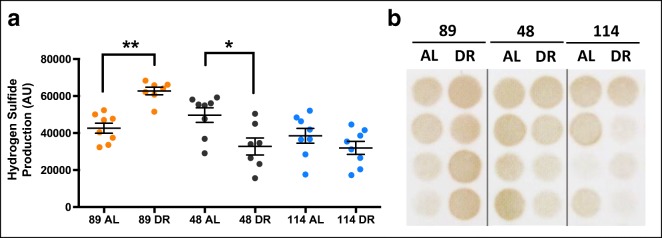
Strain-specificity exists in hepatic H_2_S production following 40% dietary restriction in female ILSXISS mice. **a** Hepatic H_2_S production levels in TejJ89, TejJ48 and TejJ114 mice on AL or 40% DR, as quantified by densitometry analysis of lead acetate assay results. **b** Representative images of lead acetate precipitates formed in the assay; darker precipitates indicate higher hepatic H_2_S production capacity. TejJ89 data in orange, TejJ48 data in black, TejJ114 data in blue. Error bars represent SEM. *p < 0.05, **p < 0.01

Using the lead acetate method to determine H_2_S production (Hine et al. 2015; Hine et al. 2017), we observed a significant genotype effect (*F* = 12.243, *p* < 0.001) but no treatment effect (*F* = 0.150, *p* = 0.701) in the liver. However, a significant genotype by treatment interaction was detected (*F* = 13.833, *p* < 0.001). Post hoc analysis indicated that H_2_S production was significantly elevated by 40% DR in strain TejJ89 (*p* = 0.005), but significantly reduced by 40% DR in strain TejJ48 (*p* = 0.031) relative to their strain-appropriate AL controls (Fig. 1a, b). In addition, H_2_S production was significantly elevated in strain TejJ89 relative to strains TejJ48 (*p* = 0.022) and TejJ114 (*p* < 0.001). This genotype effect was primarily driven by significantly elevated H_2_S production in TejJ89 under 40% DR compared with all other groups, with no differences in H_2_S production detected between ILSXISS strains under AL feeding (Fig. 1a, b). We also determined kidney H_2_S production (Fig. S1). No significant treatment effect was detected (*F* = 1.540, *p* = 0.0221), but a significant genotype effect on kidney H_2_S production was seen (*F* = 3.294, *p* = 0.047), being significantly elevated in strain TejJ89 relative to strain TejJ48 (*p* = 0.050).

### Transcript levels of H_2_S-production and -elimination proteins in ILSXISS mice following 40% DR

**Fig. 2:**
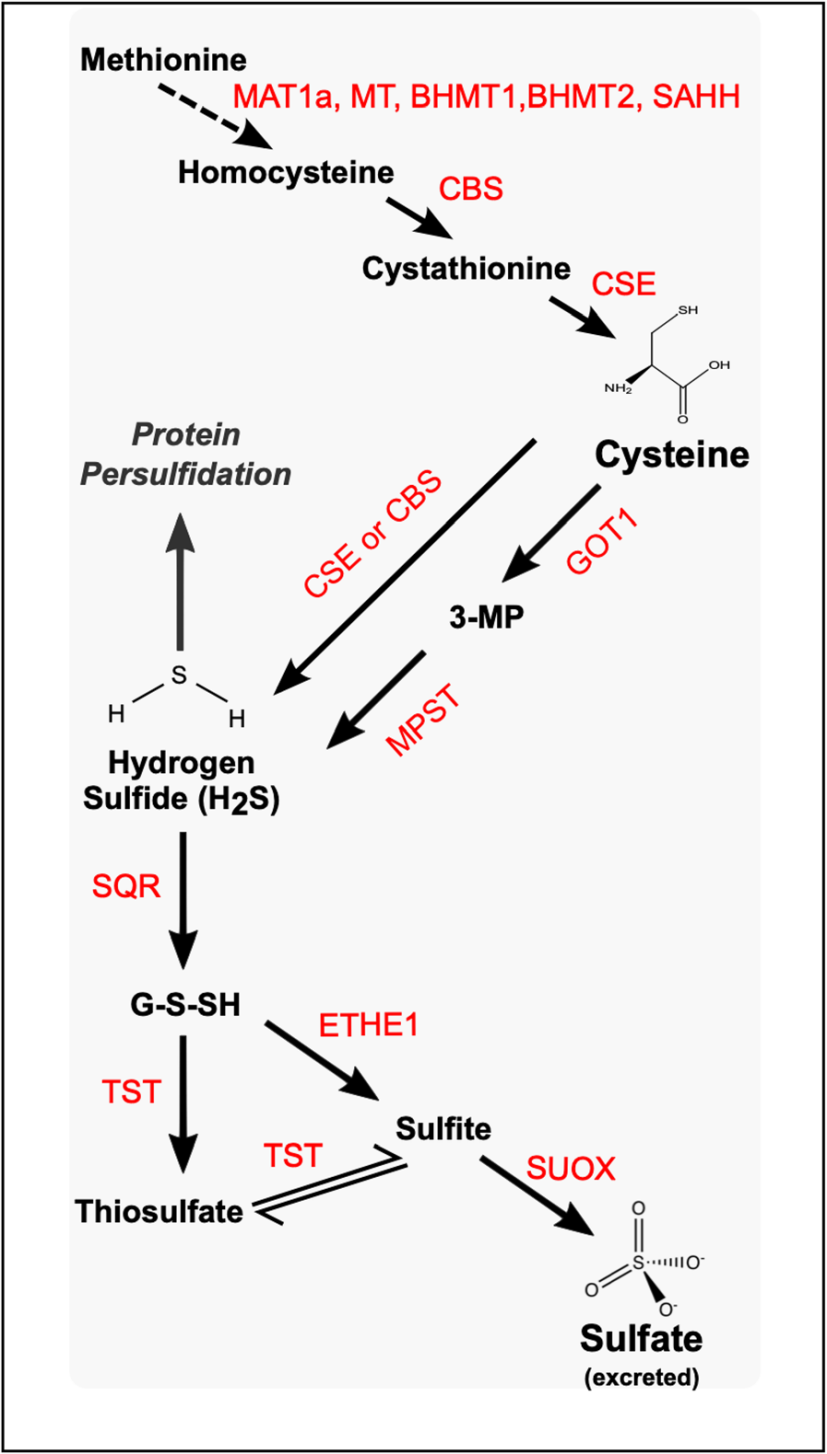
Molecular pathways involved in the enzymatic production of H_2_S from amino acid metabolism and subsequent elimination of H_2_S by components of the sulphide disposal unit. Enzymes in red. MAT1a, methionine adenosyltransferase 1A; MT, methyl transferase; SAHH, S-adenosylhomocysteine hydrolase; BHMT1, betaine-homocysteine S-methyltransferase 1; BHMT2, betaine-homocysteine S-methyltransferase 2; CBS, cystathionine-β-synthase; CSE, cystathionine-γ-lyase; GOT1, glutamic-oxaloacetic transaminase 1; MPST, 3-mercaptopyruvate sulfurtransferase; SQR, sulphide:quinone oxidoreductase; TST, thiosulfate sulfurtransferase; ETHE1, ethylmalonic encephalopathy 1 protein; SUOX, sulfite oxidase

**Fig. 3:**
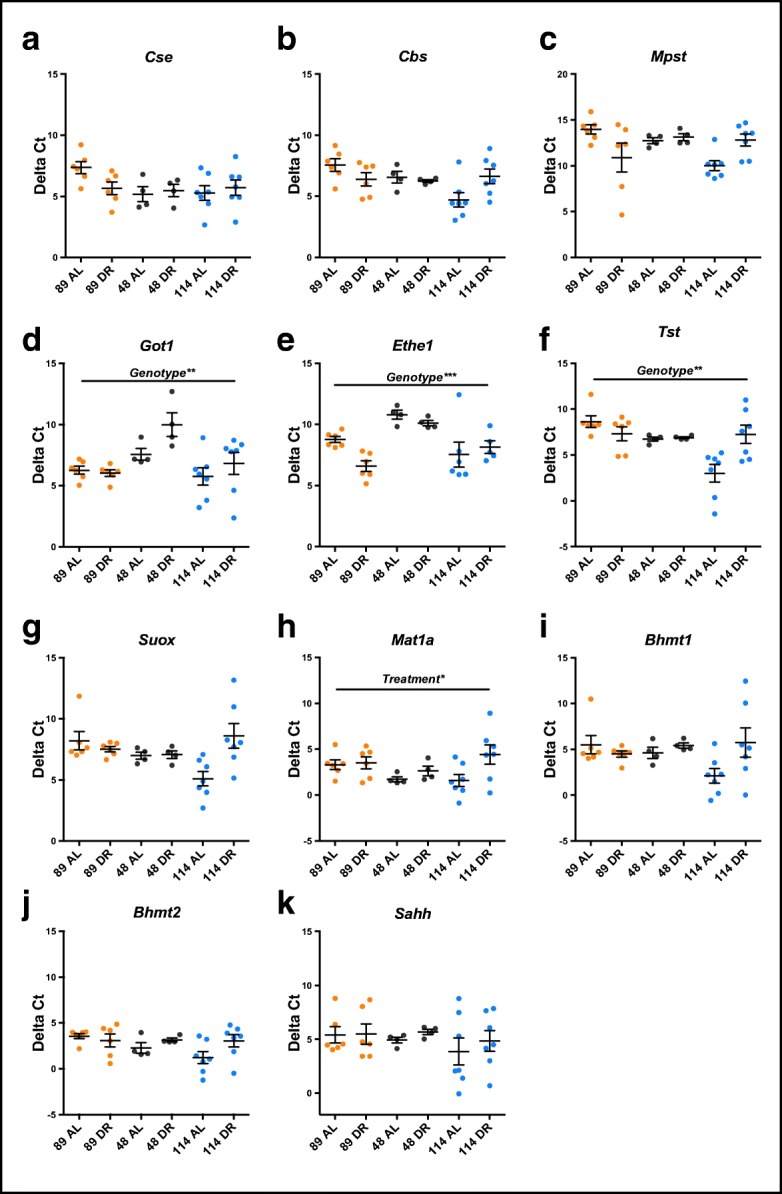
ILSXISS mouse strains exhibit differential transcriptional regulation of H_2_S-production and elimination enzymes following 40% DR. Hepatic mRNA gene expression (presented as Delta Ct values, a lower Delta Ct indicates a higher number of mRNA transcripts for that particular gene) of H_2_S-producing (**a**–**d**), H_2_S-eliminating (**e**–**g**) and methionine to cysteine conversion (**h**–**k**) genes in TejJ89, TejJ48 and TejJ114 mice under AL feeding or 40% DR. TejJ89 data in orange, TejJ48 data in black, TejJ114 data in blue. Error bars represent SEM. *p < 0.05, **p < 0.01, ***p < 0.001. Genotype (TejJ89, TejJ48 or TejJ114) and Treatment (AL or DR). See Table S1 for statistical output. *Cse*, cystathionine-γ-lyase; *Cbs*, cystathionine-β-synthase; *Mpst*, 3-mercaptopyruvate sulfurtransferase; *Got1*, glutamic-oxaloacetic transaminase 1, *Ethe1*, ethylmalonic encephalopathy 1 protein, *Tst*, thiosulfate sulfurtransferase; *Suox*, sulfite oxidase; *Mat1a*, methionine adenosyltransferase 1A; *Bhmt1*, betaine-homocysteine S-methyltransferase 1; *Bhmt2*, betaine-homocysteine S-methyltransferase 2; *Sahh*, S-adenosylhomocysteine hydrolase

In order to better understand the enzymatic pathways regulating hepatic endogenous H_2_S (see Fig. 2) across different ILSXISS strains maintained under AL or 40% DR, we determined gene expression levels of a suite of H_2_S-producing and -eliminating proteins (Fig. 3a–i). *Cse*, *Cbs* and *Mpst* transcript levels (Fig. 3a–c) were unaffected by both genotype and treatment (see Table S2 for all statistical output). However, a significant genotype by treatment interaction effect was observed for both *Cbs* (Fig. 3b, *F* = 4.737, *p* = 0.017) and *Mpst* (Fig. 3c, *F* = 6.734, *p* = 0.004), with lower expression in strain TejJ89 under AL feeding relative to strain TejJ114 under AL feeding (*p* = 0.008 and *p* = 0.024 for *Cbs* and *Mpst* respectively). *Got1* (Fig. 3d) and *Ethe1* expression (Fig. 3e) differed by genotype (*F* = 7.185, *p* = 0.003 and *F* = 10.445, *p* < 0.001 for *Got1* and *Ethe1* respectively) but not by treatment, with strain TejJ48 having significantly lower *Got1* and *Ethe1* expression relative to strains TejJ89 and TejJ114 (*p* < 0.01, in all cases). *Tst* expression levels (Fig. 3f) showed a significant genotype effect (*F* = 6.659, *p* = 0.004), with reduced expression in liver of TejJ89 mice compared with TejJ114 mice (*p* = 0.003), but again no treatment effect was detected. A significant *Tst* genotype by treatment interaction was also detected (*F* = 6.745, *p* = 0.004), with AL TejJ89 mice having significantly lower *Tst* expression levels compared with AL TejJ114 mice (*p* < 0.001), and 40% DR reducing *Tst* expression in Tej114 mice (*p* = 0.007) relative to TejJ114 controls (Fig. 3f). While no significant genotype nor treatment effect on *Suox* expression was detected (Fig. 3g and Table S2), a significant genotype by treatment interaction effect was observed (*F* = 5.694, *p* = 0.008), again with AL TejJ89 mice having significantly reduced expression relative to AL TejJ114 mice (*p* = 0.035), and 40% DR significantly reducing *Suox* expression in Tej114 mice relative to AL TejJ114 mice (*p* = 0.008). *Mat1a* (Fig. 3h) did not show any significant genotype effect (*F* = 1.069, *p* = 0.356) but was the only transcript that showed a significant treatment effect (*F* = 5.3183, *p* = 0.030), being significantly decreased by 40% DR across all ILSXISS strains. No significant genotype or treatment effects were detected for *Bhmt1*, *Bhmt2* or *Sahh* (Figs. 3i–k, Table S2).

### Protein levels of H_2_S-production enzymes in ILSXISS and C57BL/6J mice following 40% DR

**Fig. 4:**
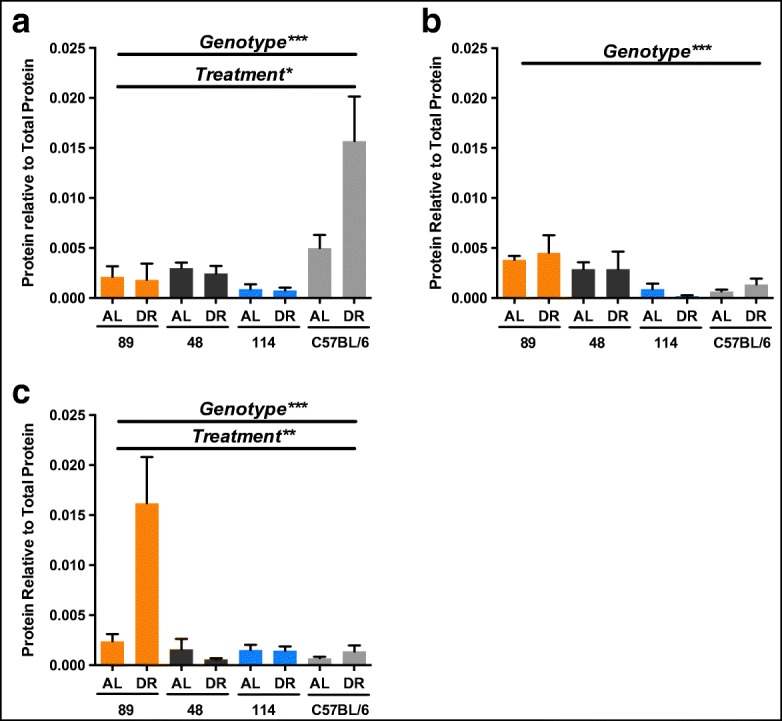
40% DR significantly increases cystathionine gamma-lyase (CSE) within the liver of C57BL/6J mice but significantly increases 3-mercaptopyruvate sulfurtransferase (MPST) within the liver of TejJ89 mice. Hepatic protein levels of CSE (**a**), cystathionine-β-synthase (CBS) (**b**) and MPST (**c**) in TejJ89 (*n* = 6), TejJ48 (*n* = 4), TejJ114 (*n* = 6) and C57BL/6J (*n* = 4) AL and 40% DR mice. TejJ89 data in orange, TejJ48 data in black, TejJ114 data in blue, C57BL/6J data in light grey. Error bars represent SEM. *p < 0.05, **p < 0.01, ***p < 0.001. Genotype (TejJ89, TejJ48, TejJ114 or C57BL/6J) and treatment (AL or DR)

Studies in C57BL/6J mice have repeatedly shown that generation of hepatic H_2_S is driven primarily through CSE and CBS, with CSE appearing to be the predominant enzymatic source (e.g. Mani et al. 2014). We subsequently compared hepatic protein levels of CSE, CBS and MPST in TejJ89, TejJ48 and TejJ114 mice with levels in C57BL/6J mice under AL feeding and 40% DR. CSE protein levels (Fig. 4a) were significantly altered by both genotype (*F* = 14.845, *p* < 0.001) and treatment (*F* = 5.559, *p* = 0.024), with a significant treatment by genotype interaction present (*F* = 5.990, *p* = 0.002). CSE levels were significantly higher in C57BL/6J mice relative to all ILSXISS strains (*p* < 0.001, in all cases), with no differences in CSE levels observed between ILSXISS strains. CSE protein levels were elevated by 40% DR but only significantly so in C57BL/6J mice (*p* = 0.002). Hepatic CBS levels were also affected by genotype (*F* = 6.451, *p* = 0.001) but not by treatment (*F* = 0.037, *p* = 0.848), with TejJ89 mice having increased CBS levels relative to both TejJ114 (*p* = 0.002) and C57BL/6J (*p* = 0.016) mice (Fig. 4b). MPST levels were significantly altered by both genotype (*F* = 12.984, *p* < 0.001) and treatment (*F* = 8.812, *p* = 0.005), with a significant genotype by treatment interaction effect (*F* = 9.848, *p* < 0.001) also observed (Fig. 4c). Hepatic MPST levels were significantly elevated in TejJ89 mice compared with all other genotypes (*p* < 0.001, in all cases). The elevated H_2_S levels observed in TejJ89 mice under 40% DR was associated with a significant elevation in hepatic MPST levels relative to TejJ89 AL mice (Fig. 4c, *p* < 0.001), but 40% DR did not alter MPST levels significantly in any other genotype compared with their appropriate AL controls. We subsequently determined hepatic MPST activity within our ILSXISS mouse strains (Fig. S2), but no genotype (*F* = 0.144, *p* = 0.707) nor treatment (*F* = 2.755, *p* = 0.081) effect was observed. Our findings indicate that significant genotype-specific differences exist in protein levels of the primary cellular H_2_S generating enzymes CSE, CBS and MPST within mouse liver (Fig. 4a–c). The increased H_2_S levels following 40% DR in strain TejJ89 was associated with an increase in MPST protein levels, but not in CSE (significantly elevated in C57BL/6J mice under 40% DR) or CBS levels, and that protein levels of these H_2_S generating enzymes were unresponsive to 40% DR in strains TejJ48 and TejJ114.

## Discussion

A reduction in the intake of calories or in the intake of macro- or micronutrients, termed here as dietary restriction (DR), is currently the most widely employed experimental intervention to modulate ageing. Indeed, DR has been shown to extend lifespan and healthspan in an evolutionary diverse group of organisms (Fontana and Partridge 2015; Speakman and Mitchell 2011), and has also been shown to provide a number of beneficial health effects in humans (Fontana et al. 2004; Fontana et al. 2007). However, it is still not understood how DR mechanistically elicits its beneficial effects. In addition, a number of studies, particularly in mice, report that the DR effect on lifespan and healthspan can vary significantly depending on genetic background (Forster et al. 2003; Goren et al. 2004; Hempenstall et al. 2010; Liao et al. 2010; Mitchell et al. 2016; Rikke et al. 2010; Swindell 2012; Turturro et al. 1999). It is believed that better understanding of the basis of this genetic variation during DR may be important if we hope to translate experimental findings from (typically) highly inbred mouse models to genetically heterogenous humans (Selman and Swindell 2018).

In this study, we investigated the potential relevance of Hydrogen sulfide (H_2_S) in DR-induced lifespan by comparing genetically distinct ILSXISS recombinant inbred mouse strains that have been reported to show significant variation in their lifespan under 40% DR, ranging from life extension to no response, through to life shortening (Liao et al. 2010; Rikke et al. 2010). A number of studies have now reported genetic or pharmacological interventions that modulate H_2_S levels can profoundly impact longevity in model organisms (Hine and Mitchell 2015; Miller and Roth 2007; Qabazard et al. 2013; Shaposhnikov et al. 2018; Wei and Kenyon 2016) and protect against age-associated dysfunction (Latorre et al. 2018; Wang et al. 2019; Zhan et al. 2018). In addition, increased hepatic H_2_S is a conserved phenotype in long-lived genetic mouse mutants (Hine et al. 2017), is increased significantly by DR in C57BL/6J and DBA/2 mice (Hine et al. 2015; Mitchell et al. 2016) and appears essential for mediating the beneficial effects of DR (Hine et al. 2015). We found that hepatic H_2_S was only elevated in female mice from strain TejJ89 under long-term 40% DR; TejJ89 is the single ILSXISS strain in our study reported to show DR-induced longevity (Liao et al. 2010; Rikke et al. 2010). In contrast, strain TejJ48 reported to be refractory to 40% DR (Liao et al. 2010; Rikke et al. 2010) showed a significant reduction in hepatic H_2_S when exposed to 40% DR. In strain TejJ114 reported to show lifespan shortening under 40% DR (Liao et al. 2010; Rikke et al. 2010), we observed no DR-associated difference in hepatic H_2_S production relative to AL mice. However, no treatment nor interaction effect was observed in kidney H_2_S production, suggesting tissue-specificity exists in the impact of DR on H_2_S production in mice, in contrast to findings (H_2_S concentration) previously reported in F344 rats under DR (Wang et al. 2016). In addition, significant strain-specificity in H_2_S production was also observed, being elevated in strain TejJ89 relative to both TejJ48 and TejJ114 in the liver and elevated in TejJ89 compared with TejJ48 in the kidney. Our findings indicate that TejJ89 mice show a similar association between increased hepatic H_2_S production and extended lifespan under DR reported in other mouse strains such as C57BL/6J and DBA/2 (Mitchell et al. 2016). Consequently, our findings do further support the premise that elevated hepatic H_2_S levels may be an important mediator of the beneficial effects of DR (Hine et al. 2015; Hine and Mitchell 2015).

To further investigate the potential processes underlying these strain-specific differences in H_2_S following 40% DR, we examined a suite of H_2_S-producing and -degrading enzymes at the transcript and protein level within ILSXISS mice. The predominately cytosolic enzymes cystathionine γ-lyase (CSE or CGL) and cystathionine β-synthase (CBS) are the main sources of H_2_S within cells (Carter and Morton 2016), and mice carrying genetic defects in these enzymes are prone to a number of pathologies (Hine et al. 2018). In particular, elevated hepatic H_2_S following DR correlates with transcript and protein levels of CSE (Derous et al. 2017; Wang et al. 2016), and similarly CSE levels are elevated in several long-lived mouse mutants (Hine et al. 2017). Perhaps surprisingly, we did not observe any genotype or treatment effects on transcript levels of *Cse*, *Cbs* or *Mpst*, although several significant genotype and genotype by treatment interaction effects (*Cbs*, *Mpst*, *Tst*, *Suox*) were detected, typically with TejJ89 AL mice having significantly reduced expression compared with TejJ114 AL mice. At the protein level, CSE was significantly elevated in C57BL/6J mice compared with all ILSXISS strains, with 40% DR further increasing CSE levels within the liver of C57BL/6J mice. However, 40% DR did not have any effect on hepatic CSE levels in ILSXISS mice. Hepatic CBS protein levels were unaffected by 40% DR across all genotypes studied. Given that both CSE and CBS levels were unaffected by 40% DR in strain TejJ89 despite the DR-associated increase in H_2_S production, we subsequently investigate 3-mercaptopyruvate sulfurtransferase (MPST). This is the third H_2_S-producing enzyme within cells but its role has been much less well characterised relative to both CSE and CBS, particularly in the context of ageing and DR. CSE and CBS primarily remain cytoplasmic under normal physiological conditions, whereas MPST can localise to mitochondria and exhibits a profound influence over mitochondrial-specific metabolism and H_2_S levels (Kimura 2014). Furthermore, while CSE and CBS work in concert to convert homocysteine into H_2_S via step-wise reactions, MPST generates H_2_S from a distinct substrate, 3-mercaptopyruvate (Renga 2011; Tao et al. 2017). We found MPST to be significantly increased in the liver of TejJ89 mice under 40% DR, although it was unaffected by DR in strains TejJ48, TejJ114 or in C57BL/6J mice. Precisely why C57BL/6J mice and TejJ89 mice appear to have distinct mechanistic routes (elevated CSE or elevated MPST respectively) to achieve the same outcome of elevated hepatic H_2_S under 40% DR still needs to be determined.

There are of course some caveats to our findings, not least that this work is highly correlational. As discussed elsewhere, the variation in phenotypic responses to DR across different mouse strains is quite broad (Mitchell et al. 2016; Selman and Swindell 2018; Swindell 2012). We examined females and then only in three strains of ILSXISS mice, albeit strains that represent the variety of lifespan responses reported in the original two studies (Liao et al. 2010; Rikke et al. 2010). We were vigilant in our choice of strains in this comparative study, choosing those that showed a similar direction of response across both studies. In addition, and as discussed in detail elsewhere (Selman and Swindell 2018), significant differences in experimental design and husbandry practices existed between the original studies. Consequently, a fuller investigation of the lifespan response to DR in ILSXISS mice and the potential relevance of H_2_S production, particularly under graded levels of DR, is warranted, but will be a major undertaking (Selman and Swindell 2018). In addition, it will also be interesting to investigate precisely how H_2_S production varies in different tissues and in different cellular locations following DR. These approaches may be made more feasible with the advent of novel chemical probes to determine H_2_S in vivo (Arndt et al. 2017; Lau et al. 2019). However, irrespective of these caveats, we have shown that endogenous H_2_S levels and associated signalling pathways differ significantly depending on genetic background in mice under both AL and DR conditions. Our data suggest that, similar to previous reports, increased H_2_S production and/or metabolism is a conserved mechanism through which DR acts to increase lifespan in mice (Hine et al. 2017; Hine and Mitchell 2015), but the precise cellular processes that regulate H_2_S production and elimination under DR appear highly strain- (and potentially tissue-) specific.

## Electronic supplementary material

ESM 1 H_2_S production levels in kidney from AL and 40% DR TejJ89, TejJ48 and TejJ114 mice, as quantified by densitometry analysis of lead acetate assay results. TejJ89 data in orange, TejJ48 data in black, TejJ114 data in blue. Error bars represent SEM. *p <0.05*. Genotype (TejJ89, TejJ48 or TejJ114). (PPTX 77 kb)

ESM 2 3-Mercaptopyruvate sulfurtransferase (MPST) activity in liver as determined by thiocyanate production capacity in AL and DR TejJ89, TejJ48 and TejJ114 mice. TejJ89 data in orange, TejJ48 data in black, TejJ114 data in blue (PPTX 67 kb)

ESM 3 RT-qPCR primer sequences (DOCX 26 kb)

ESM 4 RT-qPCR data analysis output. (DOCX 14 kb)
