# Supplementary figures and images for: Strain-specificity in the hydrogen sulphide signalling network following dietary restriction in recombinant inbred mice
Source: GeroScience. 2020 Mar 11;42(2):801–12. doi: 10.1007/s11357-020-00168-2 (PMC7205779; doi:10.1007/s11357-020-00168-2)

## Slide 1
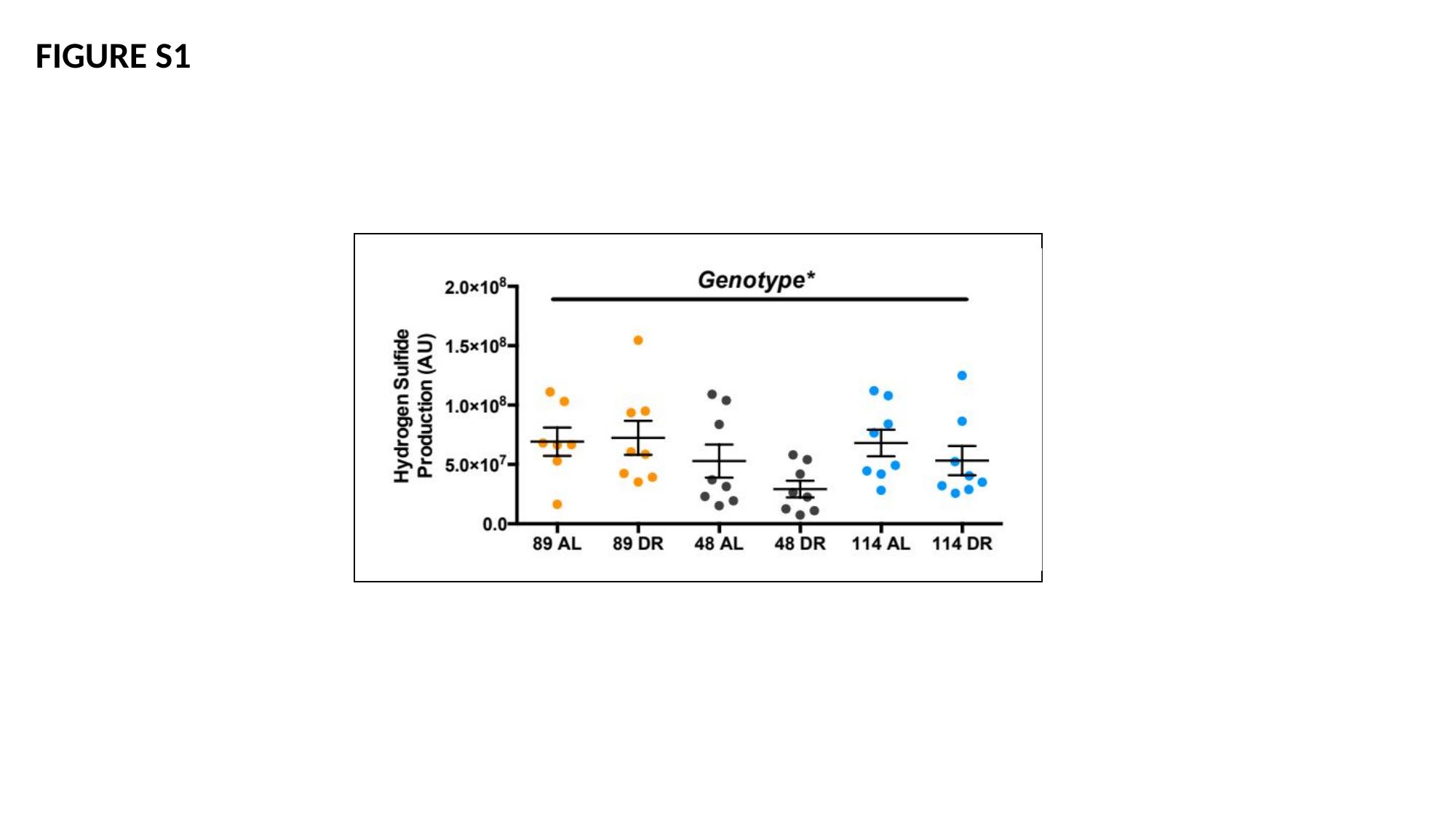

FIGURE S1

Supplement: Supplementary file 1 — H2S production levels in kidney from AL and 40% DR TejJ89, TejJ48 and TejJ114 mice, as quantified by densitometry analysis of lead acetate assay results. TejJ89 data in orange, TejJ48 data in black, TejJ114 data in blue. Error bars represent SEM. *p <0.05*. Genotype (TejJ89, TejJ48 or TejJ114). (PPTX 77 kb) [file 11357_2020_168_MOESM1_ESM.pptx]

## Slide 1
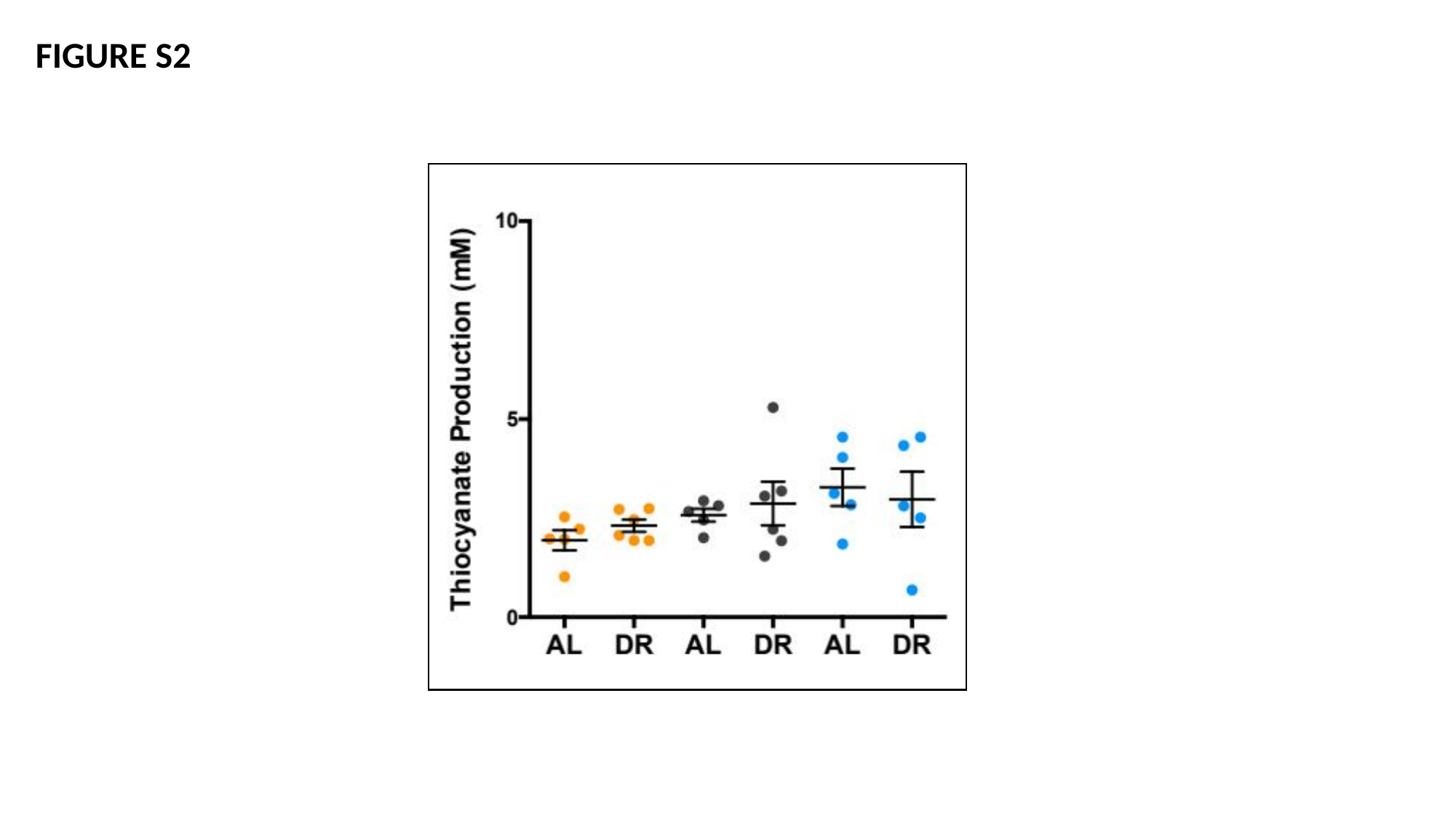

FIGURE S2

Supplement: Supplementary file 2 — 3-Mercaptopyruvate sulfurtransferase (MPST) activity in liver as determined by thiocyanate production capacity in AL and DR TejJ89, TejJ48 and TejJ114 mice. TejJ89 data in orange, TejJ48 data in black, TejJ114 data in blue (PPTX 67 kb) [file 11357_2020_168_MOESM2_ESM.pptx]
