# Supplementary material for: Strain-specificity in the hydrogen sulphide signalling network following dietary restriction in recombinant inbred mice
Source: GeroScience. 2020 Mar 11;42(2):801–12. doi: 10.1007/s11357-020-00168-2 (PMC7205779; doi:10.1007/s11357-020-00168-2)
Supplement: Supplementary file 3 — RT-qPCR primer sequences (DOCX 26 kb) [file 11357_2020_168_MOESM3_ESM.docx]

# Supplementary Table 1: RT-qPCR primer sequences

Primers were designed using the UPL Library Assay Design Centre (Roche) and BLASTn (NCBI). *Cbs*: Cystathionine-β-synthase, *Cse*: Cystathionine-γ-lyase, *Tst*: Thiosulfate Sulfurtransferase, *Mpst*: 3-Mercaptopyruvate Sulfurtransferase, *Got1*: Glutamic-oxaloacetic transaminase 1, *Ethe1*: Ethylmalonic encephalopathy 1 protein, *Suox*: Sulfite Oxidase, *Mat1a*: Methionine adenosyltransferase, *Bhmt1*: Betaine-homocysteine methyl transferase-1, *Bhmt2*: Betaine-homocysteine methyl transferase-2, *Sahh*: S-adenosyl homocysteine hydrolase.

| **Gene Name** | **Amplicon**  **(nt)** | **Intron Spanning** | **Length of Intron(s) spanned (nt)** | **Forward Primer Sequence** | **Reverse Primer Sequence** |
| --- | --- | --- | --- | --- | --- |
| *Cbs* | 84 | Y | 903 | gctgggcacactctctcac | caggcctggtctcgtgat |
| *Cse* | 78 | Y | 985 | catgctaaggccttcctcaa | ctcagccagactctcatatcctc |
| *Got1* | 97 | Y | 2350 | ttcagtttcaccggcttga | agccgcacatgttgatcc |
| *Tst* | 82 | Y | 5208 | ccagctggtggactctcg | gtggcccgagtctagtcct |
| *Mpst* | 85 | Y | 2772 | cttgccgagtgccttcac | gcctaggagatgctcagattg |
| *Ethe1* | 88 | Y | 11056 | gattccatccgctttggac | ggtcgttcaggacaaaggtg |
| *Suox* | 131 | Y | 257 | ttccacaggccatcagagt | ccatctccgagtccttgagt |
| *Mat1a* | 74 | Y | 5133 | tctgaggcgctctggtgt | cctgcatgtactgaactgttacct |
| *Bhmt1* | 96 | Y | 2419 | cggcttcagaaaaacatgg | gattctgccagtattcctttctg |
| *Bhmt2* | 83 | Y | 1758 | gacaagctggaaaacagagga | cgtgcaatgtcacaagcag |
| *Sahh* | 95 | Y | 1568 | acccagataaataccctgttgg | cagcttcacattcagcttgc |
