# Supplementary material for: Strain-specificity in the hydrogen sulphide signalling network following dietary restriction in recombinant inbred mice
Source: GeroScience. 2020 Mar 11;42(2):801–12. doi: 10.1007/s11357-020-00168-2 (PMC7205779; doi:10.1007/s11357-020-00168-2)
Supplement: Supplementary file 4 — RT-qPCR data analysis output. (DOCX 14 kb) [file 11357_2020_168_MOESM4_ESM.docx]

# Supplementary Table 2: RT-qPCR data analysis output.

# All data (raw delta CT values) were analysed using a general linear modelling (GLM) approach with genotype (TejJ89, TejJ48 and TejJ114) and treatment (AL or DR) introduced as fixed factors, and a *post-hoc* Bonferroni test employed for multiple comparisons. In all cases, non-significant interactions (p>0.05) within the GLM analyses were removed in order to obtain the best-fitting model. Significant (p<0.05) p values are indicated in bold.

| **Gene name** | **Genotype effect** | **Treatment effect** | **Genotype*treatment interaction** |
| --- | --- | --- | --- |
| *Cse* | F=2.159, p=0.133 | F=0.492, p=0.488 | — |
| *Cbs* | F=3.193, p=0.056 | F=0.110, p=0.743 | **F=4.737, p=0.017** |
| *Mpst* | F=1.619, p=0.216 | F=0.003, p=0.958 | **F=6.734, p=0.004** |
| *Got1* | **F=7.185, p=0.003** | F=2.608, p=0.117 | — |
| *Ethe1* | **F=10.445, p<0.001** | F=2.405, p=0.133 | — |
| *Tst* | **F=6.659, p=0.004** | F=2.142, p=0.154 | **F=6.745, p=0.004** |
| *Suox* | F=1.281, p=0.294 | F=2.707, p=0.111 | **F=5.694, p=0.008** |
| *Mat1a* | F=1.069, p=0.356 | **F=5.183, p=0.030** | — |
| *Bhmt1* | F=0.684, p=0.512 | F=2.172, p=0.151 | — |
| *Bhmt2* | F=2.008, p=0.152 | F=2.325, p=0.138 | — |
| *Sahh* | F=0.865, p=0.431 | F=0.614, p=0.440 | — |

*Cse*: Cystathionine-γ-lyase, *Cbs*: Cystathionine-β-synthase, *Mpst*: 3-Mercaptopyruvate Sulfurtransferase, *Got1*: Glutamic-oxaloacetic transaminase 1, *Ethe1*: Ethylmalonic encephalopathy 1 protein, *Tst*: Thiosulfate Sulfurtransferase, *Suox*: Sulfite Oxidase, *Mat1a*: Methionine adenosyltransferase, *Bhmt1*: Betaine-homocysteine methyl transferase-1, *Bhmt2*: Betaine-homocysteine methyl transferase-2, *Sahh*: S-adenosyl homocysteine hydrolase.
